# Presumed Tubercular Multifocal Choroiditis

**DOI:** 10.18502/jovr.v19i4.11199

**Published:** 2024-12-31

**Authors:** Masoud Soheilian, Pejvak Azadi

**Affiliations:** ^1^Ocular Tissue Engineering Research Center, Research Institute for Ophthalmology and Vision Science, Shahid Beheshti University of Medical Sciences, Tehran, Iran; ^2^Department of Ophthalmology, Emam Khomeini Hospital, Kermanshah University of Medical Sciences, Kermanshah, Iran; ^4^Masoud Soheilian: https://orcid.org/0000-0001-7508-426X; ^5^Pejvak Azadi: https://orcid.org/0000-0003-4776-6989

**Keywords:** Multifocal Choroiditis, Ocular Tuberculosis, Uveitis

## Abstract

**Purpose:**

To report a case of tubercular choroiditis that was initially treated for multifocal choroiditis.

**Case Report:**

A 54-year-old female patient diagnosed with multifocal choroiditis was referred to the clinic while undergoing treatment with systemic prednisone and methotrexate. The presenting visual acuity was 20/100 in the right eye and finger counting at 1 meter in the left eye. Further investigation by repeated tuberculin skin test and QuantiFERON-TB Gold test revealed tuberculosis as the probable cause of choroiditis. The patient was started on a four-drug antituberculosis regimen. Six months later, the vision improved significantly to 20/30 in the right eye and finger counting at 6 meters in the left eye, with no remaining cellular reaction.

**Conclusion:**

Tuberculosis should be considered in the differential diagnosis of multifocal choroiditis, and it is vital to perform careful history taking and thorough examinations.

##  INTRODUCTION

Despite its declining prevalence, tuberculosis remains a significant health burden, especially in less developed areas with lower vaccination coverage and higher population density.^[1]^ Ocular tuberculosis (OTB) can present with various manifestations and it should be considered in the differential diagnosis of many ocular diseases.

### Case Presentation

A 54-year-old female patient with a one-year history of bilateral vision impairment was referred to the clinic. Her medical history was unremarkable. The ocular history was positive for branch retinal vein occlusion (BRVO) and macular edema in the left eye, which had been treated with two intravitreal bevacizumab injections and macular photocoagulation. At the time of presentation, visual acuity was 20/100 in the right eye and finger counting at 1 meter in the left eye. Slit-lamp examination showed bilateral anterior chamber and vitreous reaction, which was graded +2 in the right eye and +1 in the left eye, according to the Standardization of Uveitis Nomenclature (SUN) classification.

**Figure 1:**
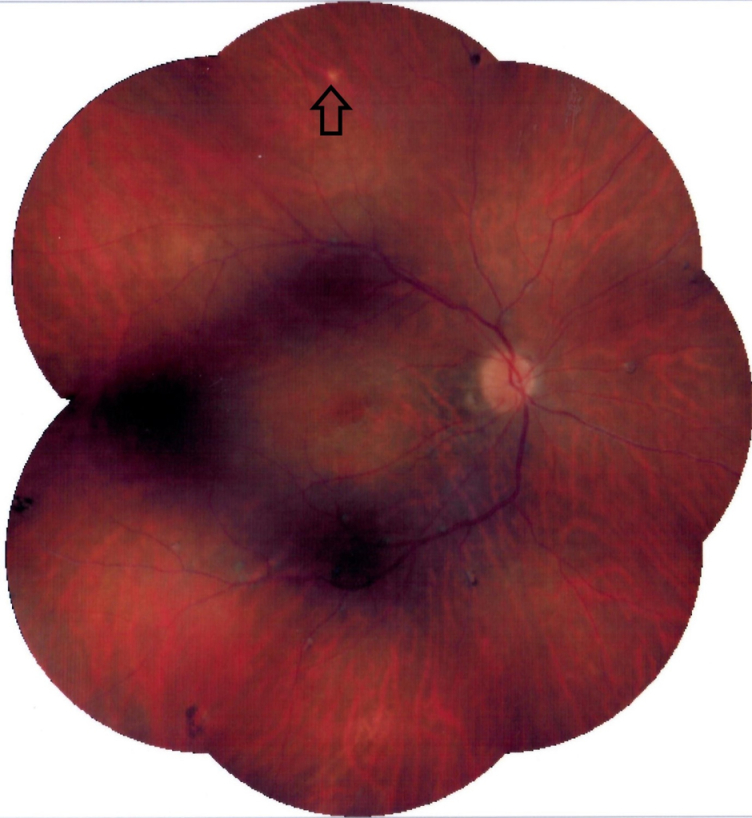
Composite color fundus photography of the right eye: Chorioretinal scars are visible in the peripapillary region and near the inferior arcade. A small creamy opaque choroiditis lesion (arrow) is present in the superior midperiphery.

**Figure 2:**
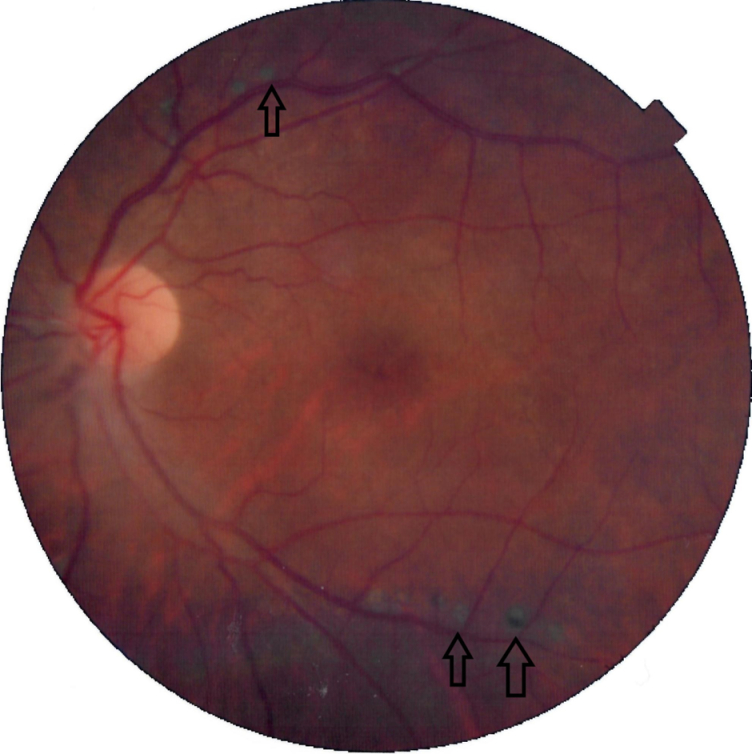
Color fundus photography of the left eye posterior pole shows the chorioretinal scars around the arcades (arrows). The more peripheral choroidal patches are not visible in this image.

**Figure 3:**
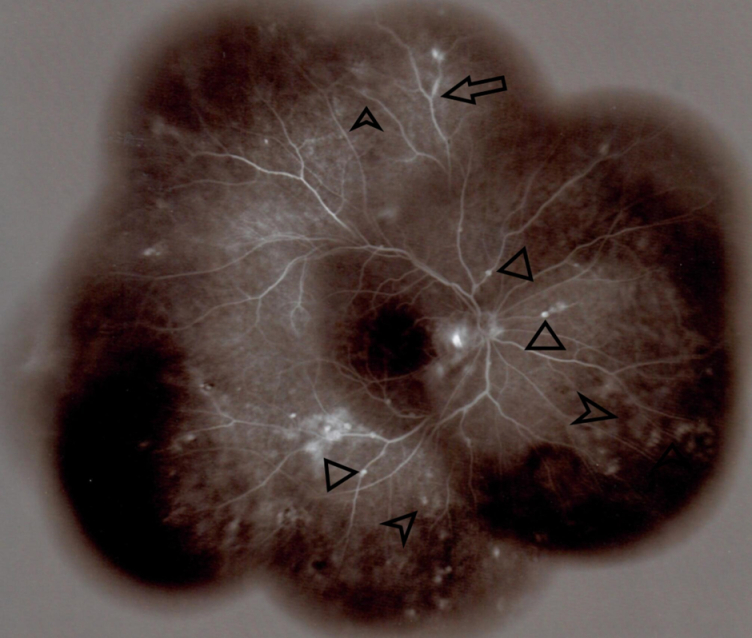
Widefield fluorescein angiography of the right eye in the late venous phase reveals small hyperfluorescent patches (pointed arrows) mainly in the anterior equatorial and midperipheral regions. Venous staining and leakage (arrows), and aneurysmal dilatation of the venules (triangles) are also evident, which are compatible with diagnosis of multifocal choroiditis and occlusive vasculitis.

**Figure 4:**
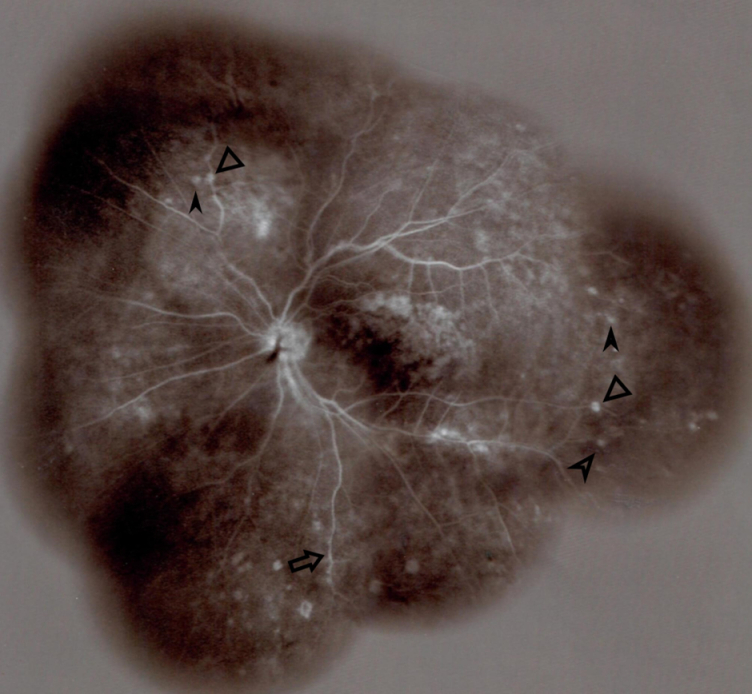
Widefield fluorescein angiography (FA) of the left eye in the late arteriovenous phase (3 minutes and 9 seconds) shows findings similar to the right eye, with the exception of hyperfluorescence in the supratemporal macular vein territory. This hyperfluorescence could possibly be due to staining of the previously treated macular photocoagulation scars and ongoing macular edema and leakage induced by branch retinal vein occlusion (BRVO).

**Figure 5:**
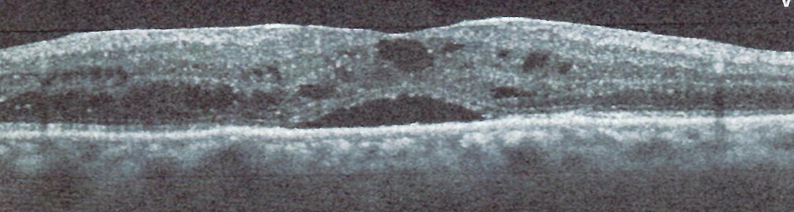
The left eye OCT shows spongy edema in plexiform layers, especially outer plexiform layer, mainly due to branch retinal vein obstruction.

**Figure 6:**
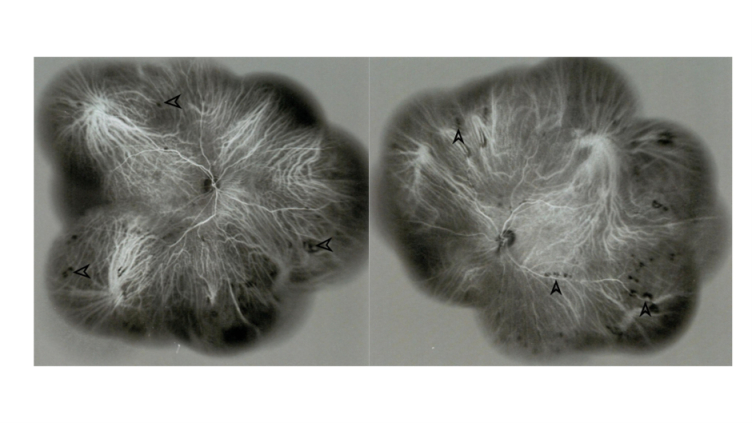
Widefield indocyanine green angiography (ICGA) in the early phase of the right and the left eyes: hypocyanescent patches (pointed arrows) are evident throughout the fundus, near the arcades, and in the equatorial and midperipheral regions. These patches are more numerous than those visible in funduscopy [Figure 1]. While some of the patches are located at the chorioretinal scars, others are more peripheral lesions that are not associated with the scars, suggesting active choroiditis lesions.

**Figure 7:**
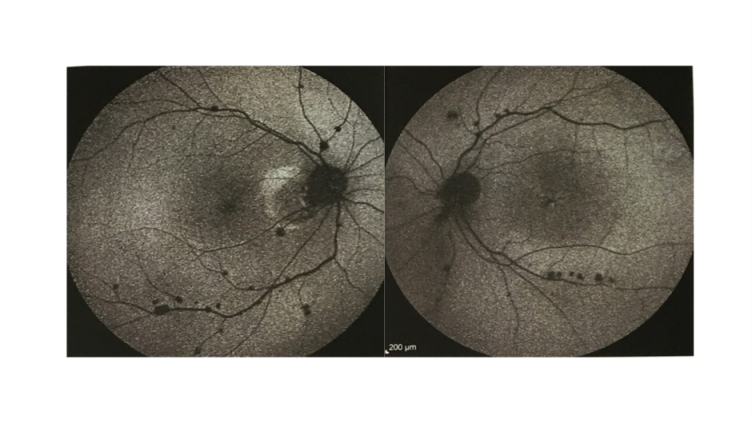
The blue autoflourescent (BAF) images of the right and left eyes showing small hypoautofluorescent spots within the areas of chorioretinal atrophy.

**Figure 8:**
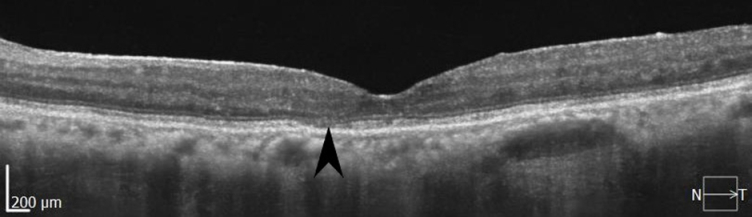
The final OCT of the left eye, taken approximately three years after the initial presentation, shows nearly complete resolution of macular edema. On the other hand, the parafoveal disruption of the ellipsoid zone (pointed arrow) explains the reduced best corrected visual acuity.

Funduscopy revealed small, creamy, opaque choroidal lesions in the midperipheral and anterior equatorial parts of both eyes. Chorioretinal scars were noted in the peripapillary region and around the arcades [Figures 1 & 2]. The results of fluorescein angiography (FA), mainly in the anterior equatorial and midperipheral parts, indicated small hypofluorescent lesions in early phases, which became gradually hyperfluorescent in later phases [Figures 3 & 4]. Venous staining and leakage as well as multiple saccular aneurysmal dilatations of the venules was also observed, which was consistent with occlusive vasculitis. The FA of the left eye showed hyperfluorescent patches at the supratemporal macular vein territory. This finding was compatible with the staining of previously performed macular photocoagulation scars and macular BRVO-induced edema and leakage [Figure 4]. Macular edema was also seen in the optical coherence tomography (OCT) images [Figure 5]. In indocyanine green angiography, hypocyanescent patches were seen around the disc, arcades, and in the periphery in the early and mid phases. These patches were more numerous than those seen in the funduscopy, and while some of them were compatible with fundus scars, the more peripheral ones could represent choroidal patches [Figure 6]. These patches appeared as hypoautofluorescent patches in the blue autofluorescence images [Figures 7].

Diagnosed as multifocal choroiditis, systemic steroid (prednisone 15 mg/day) and methotrexate (15 mg/ week) were prescribed for the patient, but no significant improvement was achieved. She was followed up regularly for about three months thereafter. While the patient's vision and signs of ocular inflammation did not improve, further history taking revealed that the patient had a long history of exposure to an individual with tuberculosis at home. Therefore, despite a previously negative tuberculin skin test (TST), we repeated the test and obtained a significantly positive result of 42 millimeters. For a more accurate diagnosis, the more specific QuantiFERON-TB Gold+ test known as interferon-gamma release assay (IGRA) was performed and the result was positive. The chest X-ray findings were not indicative of pulmonary involvement, but consultation with an infectious disease specialist strongly suggested the possibility of OTB. Therefore, the methotrexate was discontinued, prednisone was lowered to 10 mg/day, and the standard nine-month antituberculosis treatment (ATT) regimen comprising isoniazid, rifampicin, pyrazinamide, and ethambutol was started. After two months, pyrazinamide and ethambutol were discontinued. The patient's symptoms and signs improved significantly after the ATT regimen and steroid treatment while the patient was not receiving any systemic drug anymore. At the final visit, three years after the first visit, the patient's vision improved to 20/30 in the right eye and 20/40 in the left eye, and there was no anterior chamber reaction or vitritis. The OCT results showed nearly complete resolution of macular edema, with only minor disruption of the parafoveal ellipsoid zone [Figure 8].

##  DISCUSSION

As one of the top 10 causes of death worldwide, tuberculosis has been a major health problem throughout much of human history. In 2017, tuberculosis was the leading infectious organism, accounting for about 1.6 million deaths.^[1]^ While tuberculosis remains mainly a pulmonary disease with decreasing incidence, its extrapulmonary manifestations occur more frequently and their incidence has not reduced considerably.

OTB is associated with significant morbidity^[2]^ and could present with different manifestations. Of these, the most important one is tubercular uveitis (TBU), which could present as anterior uveitis, intermediate uveitis, posterior uveitis, or panuveitis.^[3]^ The tendency of aerobic bacillus *Mycobacterium tuberculosis* to target the choroid, which has a high oxygen delivery, explains why posterior uveitis is the most common manifestation of TBU.^[3]^ Each suspected patient should be thoroughly investigated for TB due to diversity of presentations, difficulty in obtaining adequate samples to confirm the disease, often associated low bacterial load,^[4]^ and absence of concomitant pulmonary TB in most cases of OTB.^[2]^ According to the Standardization of Nomenclature for Ocular Tuberculosis- Results of Collaborative Ocular Tuberculosis Study (COTS) Workshop, uveitis could be considered tubercular in origin based on positive immunological tests (e.g., Mantoux test or IGRA) and radiological evidence (e.g., chest computerized tomography indicating old/healed tuberculosis), even in the absence of confirmatory biopsy, histopathology, or polymerase chain reaction tests.^[7]^

Tubercular choroiditis (TBC) has different clinical phenotypes depending on genetic factors, immune system status, and endemicity. Primarily, it affects the choroidal stroma or choriocapillaris and often presents as a mild to moderate bilateral, insidious condition with or without associated vitritis.^[5]^ The most common manifestations of TBC include tubercular serpiginous-like choroiditis, tuberculoma, and tubercular multifocal choroiditis.^[6,7]^ However, it could also present as tubercular focal choroiditis, ampiginous-like choroiditis, or APMPPE- like choroiditis. Agarwal et al showed that both the mycobacterium activity and the immune system activity influence TBC presentations.^[8]^ They reported that, on a spectrum, the mycobacterium activity is mostly involved in choroidal tuberculoma, whereas the immune system plays a greater role in the APMPPE-like choroidal tuberculosis, with other presentations falling in between.^[6]^

Our patient's poor response to immunosuppressive treatment and her significant improvement following ATT suggest the more prominent role of mycobacterium activity in this case.^[9]^ Meanwhile, some contradicting reports have shown that adding systemic corticosteroids may increase the risk of treatment failure.^[10,11]^ Therefore, physicians should postpone systemic corticosteroids until after the initiation of ATT, unless there is a high risk of complications secondary to intense inflammatory reactions.^[10,11]^

It is also crucial to consider the role of clinical suspicion in the tuberculosis endemic areas, especially when there is a history of long-time house contact. Therefore, despite the initial negative result of the TST, it was performed again which turned positive and it was further confirmed by the IGRA test. Since the first negative TST could be false negative,^[12,13]^ the booster effect should also be considered, in which the previously infected older patient's immune system ability to react to the tuberculin test declined gradually, so the first TST turned negative, but it stimulated the immune system, resulting in a boosted positive reaction to the second TST.^[12]^ On the other hand, in people over five years old who have already received the Bacille Calmette-Guérin (BCG) vaccine, the Centers for Disease Control and Prevention recommends the TB blood test IGRA for screening ^[12]^ and combining it with TB skin tests to improve sensitivity and specificity.

Choroiditis is the most common cause of macular edema in patients with OTB, but it should also be considered in the differential diagnosis of retinal vasculitis and aneurysmal dilatations of retinal arteries.^[14,15]^ In the present case, the patient had developed BRVO most probably due to OTB-related occlusive vasculitis, and she was treated successfully with a multidisciplinary approach including ATT, immunosuppressants, and intravitreal anti-VEGF injections.

In summary, in regions with high prevalence of tuberculosis, especially developing countries, TB should be considered in the differential diagnosis of multifocal choroiditis. Besides, it is imperative to conduct careful history taking and comprehensive examinations to ensure accurate identification and treatment.

##  Financial Support and Sponsorship

None.

##  Conflicts of Interest

None.
